# Recent Progress in the Application of Natural Language Processing for Infectious Disease Detection and Surveillance in Clinical Data

**DOI:** 10.1055/s-0046-1816632

**Published:** 2026-11-03

**Authors:** David Martinez, Vlada Rozova, Curtis Murray, Wei Han, Anna Khanina, Lawrence Cavedon, Karin Verspoor

**Affiliations:** 1 School of Computing Technologies, RMIT University, MelbourneAustralia; 2 Centre for Digital Transformation of Health, University of Melbourne; 3 School of Computing and Information Systems, University of Melbourne, MelbourneAustralia; 4 National Centre for Infections in Cancer, MelbourneAustralia; 5 Department of Infectious Disease, Peter MacCallum Cancer Centre, MelbourneAustralia; 6 Sir Peter MacCallum Department of Oncology, University of Melbourne, MelbourneAustralia

**Keywords:** Natural Language Processing, Biosurveillance, Electronic Health Records, Communicable Diseases

## Abstract

**Objectives** : In this review, we examine the application of natural language processing (NLP) methods to clinical documents from electronic health records for the detection and tracking of infectious diseases.

**Methods** : We searched academic databases using artificial intelligence-assisted tools to capture all relevant literature from 2022 onwards.

**Results** : Our review covers the large body of research around COVID-19, other human infectious diseases, new and emerging diseases, veterinary medicine, general solutions for clinical NLP, and resources and datasets.

**Conclusion** : We observe that large language models are being successfully used in recent work for a variety of tasks, including named entity recognition, relation extraction, and text classification.

## 1. Introduction

Infectious diseases pose a serious threat to global health and, as seen recently during the COVID-19 pandemic, may require significant healthcare resources 1 . Early detection of infection outbreaks is key to their successful management, enabling rapid intervention strategies that aim to prevent widespread outbreaks. This early detection is typically supported through disease surveillance strategies based on finding and recording data about infection cases [ 1 ]. Data sources for public health surveillance vary in their purpose and coverage; from disease-specific case reports and population surveys, to site-wide electronic health record (EHR) systems, to social media, news reports, and research articles [ 2 ]. Of those, EHRs have the advantage of capturing relevant risk factors for a wider cohort of patients, representative of the local population [ 3 ]. Implementation of detection algorithms within EHR systems promises rapid identification of individual cases and outbreaks.

The majority of clinical information in EHRs is documented as free text, with key data elements often not readily available for analysis [ 4 5 6 ]. This challenge has driven extensive effort to develop tools to extract clinical concepts, standardise unstructured data, and classify free-text documentation. In this review, we focus on natural language processing (NLP) techniques applied to clinical data, with a particular emphasis on EHR data, to support the surveillance of infectious diseases. The NLP approaches applied in this context have grown increasingly sophisticated, evolving from dictionary- and rule-based methods, models based on term frequencies and word embeddings, to transformer-based models, and, more recently, generative large language models (LLMs).

A comprehensive survey of NLP applications for general outbreak detection was presented in [ 2 ]. In their survey, the authors identify two distinct bodies of work: (i) health mention classification, which involves selecting relevant text from a large volume, and (ii) health event detection, which involves predicting an epidemic event (defined as an outbreak over time or over a geographic region) from texts. Health mention classification can occur at the level of an individual, while a health event requires aggregation across individuals. In the context of infectious disease surveillance from clinical data, of these we identify health mention classification as the most relevant NLP task. The earlier survey further identified only four works addressing the use of medical texts [ 7 8 9 10 ]: all of these targeted surveillance of syndromes (categories of illness) rather than the tracking of specific diseases, and the texts considered were derived exclusively from emergency departments.

This survey and other previous work have identified specific challenges in the extraction of information from EHRs, such as the large presence of abbreviations and acronyms, the use of incomplete sentences, the lack of training and evaluation data due to privacy concerns, and the changing nature of the terminology and the difficult integration with existing ontologies [ 2 ]. These issues and the large variability in the types of unstructured data found in EHRs make this domain particularly difficult for applying the latest innovations in LLMs and generative Artificial Intelligence (AI) [ 11 ]. For infectious diseases specifically, identification may rely on a diversity of tests (such as CT scans or tissue sample reports), which have their own reporting procedures and varying characteristics. An added difficulty is that these reports do not provide a diagnosis or result, but rather collect all the relevant information from the test, for a future diagnosis. This makes the text dense with information, and vague with regards to any specific disease.

The dynamic characteristics of this domain are also reflected in the emergence of new diseases that require new terminology and relations to existing terms. This is particularly true in the context of infectious diseases, which can emerge suddenly and present in novel ways. The COVID-19 pandemic highlighted this need and spurred renewed interest in the use of NLP methods for infection surveillance. This review adds to the body of knowledge by examining the growing breadth of research in this space and summarising how methods are evolving from traditional rule-based approaches to adopting the latest NLP techniques. In order to achieve high recall of the literature, we perform a thorough screening of recent literature, and identify current trends in this field.

We do not include infectious disease research focused on other sources of information, such as social media or news reports, since challenges faced when dealing with this source are different: noisy data, self-reporting bias, non-technical language and slang, etc. [ 12 ]. We also consider formal reporting systems such as Program for Monitoring Emerging Diseases (ProMED-mail) [ 13 ] out of scope, as these do not rely directly on monitoring of data collected in the normal course of clinical care. While acknowledging that these are important sources for a multi-pronged approach to AI-enabled epidemic surveillance [ 14 ] and can provide useful resources for NLP research [ 15 ], we chose to focus on the challenges of processing EHRs since this is a primary source of detection, and there already exist a large variety of types of data and trends in this area. In cases where information from different sources was applied, we include it only if NLP on EHRs was a component of the architecture.

Following our screening, we structure this review by types of infectious diseases, describing applications to COVID-19, other known human infectious diseases, and emerging and new diseases. We briefly review applications and describe clinical NLP research that is not specific to infectious diseases, but aims to develop general solutions for EHRs. Finally, we outline some of the publicly available datasets that can be used to develop new methods for infection surveillance.

## 2. Methods

Our literature review combined traditional academic search approaches with AI-assisted tools, with the aim of capturing recent developments in surveillance of infectious diseases through the use of NLP on clinical records. We did not define a specific clinical research question but rather were interested in identifying and summarising research related to this topic. Hence, our review is a general literature survey rather than a systematic assessment of evidence related to a specific question. We conducted searches using academic databases (Google Scholar, PubMed), article repositories (ACL anthology 2 , arXiv, MedRxiv, PubMed Central), workshops [ 16 17 ] and special issues of journals, review papers [ 11 ], [ 18 19 20 21 22 23 24 25 26 27 28 ], making use of the AI tools Google Notebook LM 3 and EXA 4 .

Our search strategy combined different sets of terms to retrieve articles that fit the scope across the following four dimensions. These are the applied terms for each category:

(i) Surveillance context: *surveillance, biosurveillance*
(ii) Methodological approach: *NLP, LLM, text mining*
(iii) Disease focus: *infectious diseases, COVID, COVID-19*
(iv) Data source: *EHR, clinical records, patient records*

Different term combinations were used for the different article repositories, to make the screening manageable. The search was conducted in December 2024, and the initial inclusion criteria were articles from 2022 that applied NLP on EHRs for the purpose of processing infectious diseases. However, given limited results, this criterion was relaxed to include NLP for EHR that did not specifically target infectious diseases, but which was considered generalisable and potentially impactful in this area.

The main source of publications was Google Scholar, where the terms “surveillance”, “NLP”, and “infectious diseases” returned approximately 1,200 hits, of which less than 100 were retained after screening of abstracts. The other bibliographical sources provided less than 100 hits each, and after screening less than 50 articles were kept (removing duplicates). To complement the traditional keyword search, we used EXA to perform search from natural language queries that combined the category terms with relevant restatements of our scope, to help identify papers that may have been missed through exact keyword searches.

Notebook LM was a useful tool to aid the screening, allowing to query targeted information within the papers, and providing easy access to relevant paragraphs in the full text of a paper when the abstract was not enough. To expand our reach, we manually checked articles from the citation networks of the original articles (“snowball” search [ 29 ]).

## 3. Results

In our review of the recent literature involving the use of NLP in infectious disease research, we identified several distinct areas. We initially present the large body of research around COVID-19, which had an enormous global impact recently. We found that NLP research in this area targeted multiple tasks: basic detection of positive reports, determination of symptoms and attributes (including temporal characteristics and risk factors), symptom-based screening to prioritise testing, conversion of clinical notes into structured data, processing of different languages, image processing and report generation.

Other human infectious diseases have received less attention, and apart from identifying positive reports (e.g., for invasive fungal infections or for pneumonia), recent work has focused on surveillance, by detecting outbreaks via the terms identified in EHRs over time. These articles tend to deal with smaller datasets, which affects the choice of NLP models.

Another trend that we have identified is research on new and emerging diseases. These require different techniques, such as grouping of positive cases to detect deviations from known disease patterns. Hierarchical classifiers are also built to predict new infections, and systems are evaluated on retrospective identification of new diseases, as well as on manually annotated data.

Veterinary medicine and zoonotic diseases (infectious diseases that can be transmitted between other vertebrates and humans) present their own challenges and possibilities. The easier access to animal medical records has motivated research into generative AI by building different LLMs and fine-tuning. The contribution of NLP for tasks such as disease exposure pathways is also explored in this domain by relying on open text questionnaires.

For completeness, we include a section on clinical NLP as a general task, which is a promising research effort to advance research targeted to infectious diseases. These projects aim to develop general solutions for EHRs, and they are potentially applicable to extract relevant information for infectious diseases.

Table 1 summarises the main articles discussed in this section according to their target disease and characteristics. We will expand these articles in the following sections, and explain their contributions to the field. Finally, we discuss articles that present datasets and resources for this domain. We list these in Table 2 for reference.

**Table 1:** Summary of articles according to disease, NLP tasks, and NLP methods. DD: Disease Detection, EE: Entity Extraction, PM: Predictive Modelling.

**Table 2:** Infectious Disease Related Datasets and Resources, reported as T = text, I = image, N = numerical, Typh. = Typhoid, Varic. = Chickenpox, Imp. = Impetigo, Deng. = Dengue, FI = Fungal Infection, CC = Common Cold, Pneu. = Pneumonia, Jau. = Jaundice, Mal. = Malaria, UTI = Urinary Tract Infection.

1 https://physionet.org/content/mimic-iv-note/2.2/

2 https://physionet.org/content/chest-imagenome/1.0.0/

3 https://www.kaggle.com/datasets/raddar/chest-xrays-indiana-university

4 https://www.health.gov.au/resources/collections/nndss-public-datasets

5 https://www.kaggle.com/datasets/niyarrbarman/symptom2disease

6 https://www.kaggle.com/datasets/artemminiailo/medicalconversations2disease

### 3.1. COVID-19

The emergence of COVID-19 in 2020 led to many NLP research efforts in the area, mainly focusing on scientific articles and social media to identify trends. The workshops on NLP for COVID-19 held at ACL 2020 and EMNLP 2020 [ 16 17 ] and the review article from [ 51 ] compile and summarise some of this early work. However, there was limited work on NLP applied to EHRs at that time; this has grown as the value of analysing clinical texts for surveillance and diagnostic support in this context has become apparent.

Early research focusing on clinical data aimed to identify positively diagnosed COVID-19 patients for surveillance purposes. One group automated the analysis of free text documents in the US Department of Veterans Affairs Clinical Data Warehouse to classify documents as indicating that a patient had a positive COVID-19 diagnosis [ 52 ]. This work relied on a list of keywords to find documents mentioning COVID-19 or a synonym, and applied the ConText algorithm [ 53 ] and other strategies in a rule-based NLP pipeline to determine a positive diagnosis. Performance was estimated at 82.4% precision and 94.2% recall based on a manual review of the data warehouse; impressively, the algorithm enabled the detection of a large number of COVID-19 cases lacking corresponding laboratory data, 36% of the total number.

Several works have leveraged NLP methods to characterise the clinical presentation of COVID-19. Rule-based systems for extracting symptoms of COVID-19 from EHR data were described in [ 30 ], with Wang *et al.* adapting an earlier NLP tool [ 31 ], CLAMP [ 54 ] to identify eight attributes of the infection (body location, severity, temporal expression, subject, condition, uncertainty, negation, and course) from patient records. A similar approach was adopted in a retrospective study of over 85k emergency department encounters [ 32 ] using the cTAKES [ 55 ] NLP pipeline. This study found that NLP methods were more accurate for identifying patient encounters with COVID-19 symptoms than reliance on traditional International Classification of Diseases (ICD) coding. This demonstrates the value of transforming unstructured text collected in the standard course of care into more structured features. The task of identifying symptoms and risk factors of COVID-19 in EHR data of patients tested for the virus has also been tackled through weakly supervised estimation methods building on ontology-based labelling [ 33 ], illustrating the use of more sophisticated algorithms.

Such features have formed the basis of methods aiming to support symptom-based screening to prioritise testing, although presenting symptoms alone may not provide sufficient information to discriminate between different types of viruses [ 30 ]. Prediction of future COVID-19 positive cases has also been proposed based on the presence of disease-specific symptoms and their combination [ 34 ]. This work adopted the SciBERT pre-trained language model [ 56 ] to detect mentions of symptoms in 26 categories, and then applied statistical analysis to identify symptoms enriched in COVID-positive versus negative patients. COVID-19 case reports have also been analysed using NLP to extract clinical factors and social determinants of health, supporting data-driven public health research [ 57 ].

A 2022 project on COVID-19 applied NLP to a large observational study of 350,000 patients in a large integrated health care system, also focusing on clinical characterisation, including symptom onset date [ 36 ]. Dictionary- and rule-based analysis was used to identify a substantial number of symptomatic patients that had been defined as asymptomatic based on structured data alone, and relevant symptoms could be detected earlier. In [ 35 ], joint recognition of epidemiological entities and social relations from Chinese-language case reports is performed with a multi-task neural network and a BERT [ 58 ]-based pre-trained language model, achieving 97.7% F1 for the recognition of basic patient information, 78.8% F1 for infection contact events, and 45.7% F1 for the identification of specific social relations (such as familial or work relationships). By connecting individuals together based on this data, an epidemiological knowledge graph and a corresponding inference engine were also built to uncover the infection modes, sources and pathways.

Telehealth visits were prevalent during the pandemic, and typically documented only in a free text note, leading to the use of NLP to convert the notes into structured data [ 37 ]. Specifically, a mix of named entity recognisers based on various technologies including both dictionary lookup and machine learning (ML) were deployed to capture 19 categories of information, ranging from symptoms and risk factors to lab tests and medications. These could then be used as features for a model predicting a SARS-CoV-2 test result, as well as to support the optimisation of pooled testing [ 37 ].

More recent work has embraced advances in NLP modelling, adopting large-scale pre-trained language models and encoder-decoder models for generation. One study builds BERT-based classifiers for patient-initiated EHR messages, fine-tuning DistilBERT with high performance [ 38 ]. Three target classes were considered: confirmation of COVID-19 infection (COVID-19 positive), mention of COVID-19 without indication of a positive test result (COVID-19 other), or unrelated to COVID-19 (non–COVID-19). Upon deploying this classifier on over 187k messages, 3048 COVID-19 positive messages were identified, with nearly 98% of these not otherwise documented as SARS-CoV-2 positive in the EHRs. This highlights another way in which NLP can be used to enrich data for public health surveillance.

Liu *et al.* [ 39 ] built Med-MLLM, a pre-trained multi-modal model based on both image and text datasets, including health records from MIMIC-CXR and MIMIC-III The model was fine-tuned for the tasks of COVID-19 image report generation, disease diagnosis, and survival prediction. It was tested retrospectively, on early COVID-19 datasets; and prospectively, on data for a then-new variant, COVID-19-Omicron. Strong performance was observed across these tasks, over a number of diverse datasets, even in scenarios with very limited labelled data. The report generation task was the most challenging, but the system substantially outperformed prior results across English/Spanish/Chinese report datasets.

Overall, NLP has been broadly applied to transform COVID-19-related clinical data into structured data not available elsewhere in the EHRs, identifying both direct mentions of the infection and relevant symptoms, with clear positive value for detection and particularly surveillance of COVID-19 infections. In addition, the identification of symptoms and risk factors in patient records has supported the development of predictive models for diagnosis and outcome of patients suffering from the disease, with prospective value.

### 3.2. Other Known Diseases

Only three standalone research articles and a survey covering diseases other than COVID-19 were found for this section. The articles addressed Invasive fungal infections (IFIs), Influenza-like illnesses (ILIs), and Pneumonia. A common characteristic of the applied NLP techniques is the use of entity or concept recognition as an intermediate step to classify reports. Different approaches are applied on top of entity and concept-level extraction, based on manual rules and machine learning (Logistic Regression and Gradient Boosting) to classify them as positive/negative for the target disease. The methods are applied to a variety of EHRs: cytology and histopathology reports, patient care notes, emergency department notes, radiology reports, and discharge summaries. The datasets tend to be smaller and annotated for each specific task.

In [ 11 ], a systematic review was conducted on the use of NLP and LLMs in the diagnosis and prediction of infectious diseases. They identified 15 articles (published between 2005 and 2023) on these topics. The articles cover a variety of diseases, and we identified only two that fall within our scope, since most articles are prior to 2022: [ 40 ] for IFIs, and [ 38 ] for COVID-19. Some of the studies reported in the systematic review [ 47 49 ] apply LLMs in a predictive or generative capacity, rather than to support text analysis or extraction. We chose to include these in a separate section, since they are not strictly within our scope.

IFIs are studied in [ 40 ] for automated surveillance over cytology and histopathology reports. The authors apply dictionary-based NLP and ML techniques (Logistic Regression and Gradient Boosting) to identify IFI-positive reports, achieving high precision and recall. The system is part of a broader project to develop a system for automated IFI surveillance. The manually annotated dataset of 283 patients was publicly released as the Cytology and Histopathology IFI Reports corpus (CHIFIR) [ 59 ].

Other recent surveillance work has been applied to ILIs [ 41 ], with the application of the rule-based Topaz parser [ 60 ] to identify clinical findings in free-text patient care reports. These normalised terms are used as features to model the likelihoods of diseases of interest. In turn, these values are utilised to compute the expected number of patients of each disease, and the p-value of the data per day in a time series. This framework allows the authors to detect outbreaks for the target and even unknown illnesses, when the probability of the data deviates from the expected. Their models presented a high correlation with known ILIs. As one of the limitations of their framework, the authors mention the use of Topaz for NLP, and they propose MetaMap [ 61 ] for future work, in order to extract a larger variety of findings associated with diseases.

Previous work on surveillance of ILIs has relied on NLP applied to Emergency Department (ED) notes. In [ 10 ], the authors built a model based on lexical divergences in the texts accumulated over consecutive periods of time. They found that by relying on some supervision (to identify distinguishable high-frequency terms), and utilising the Jensen-Shannon Divergence (JSD) to measure lexical change, they can detect outbreaks for the three syndromes in their study: ILI, Acute Respiratory, and Diarrhea.

Pneumonia is targeted by a recent rule-based NLP system [ 42 ]. It leverages the ConText algorithm [ 53 ] to recognise positive cases of pneumonia from three different types of clinical notes (ED notes, radiology reports, discharge summaries) from an EHR collection from Veteran Affairs. The authors found moderate to high performance in the recognition task using all note types. The system was then evaluated for interoperability by measuring performance over the same types of records from an EHR collection obtained from the University of Utah, USA. Performance levels were maintained except for a drop in precision observed using discharge summaries. Error analysis showed that one contributing explanation for the drop was when a discharge summary discusses an initial (incorrect) clinical hypothesis within the discharge text.

Overall, we observe that there are few recent studies for known infectious diseases other than COVID-19, and the data sources are different. Apart from CHIFIR, the studied datasets in this section are not publicly available, although [ 41 ] and [ 42 ] make the code available for application to other datasets.

### 3.3. Emerging and Unknown Diseases

According to the WHO, emerging diseases are those that appear in a population for the first time, or that may have existed previously but are rapidly increasing in incidence or geographic range 5 . In recent years, there has been growing research on the early detection of outbreaks of such diseases, and NLP methods are often required in workflows. We identified three papers that fit our criteria for this section. The NLP methods applied target the detection of emerging diseases from ED data (complaints and patient care reports), and complete EHRs. The techniques involved topic models, hierarchical classifiers (TextCNN combined with Multi-Head Attention, and clustering), and deviations from probabilistic models of known diseases.

The need for pre-syndromic surveillance, which detects biothreats with rare or previously unseen symptomology has been discussed [ 44 ]. In this work, the authors implement and evaluate a system that learns newly emerging syndromes from free-text ED chief complaints, and can identify case clusters among subpopulations. The core of the method uses topic models, by extending Latent Dirichlet Allocation (LDA) to compare pre-learned static topics with emerging topics that differ from them. The authors call their approach Contrastive LDA, and they evaluate relevant cluster detection over historical chief complaint data from New York City (NYC), involving more than 28 million ED cases from 53 NYC hospitals during 2010–2016. They also include further experiments to evaluate the use of practitioner feedback and the retrospective detection of COVID-19 clusters in NYC. Their results show that the deployed system (i) significantly improves a keyword-based baseline, (ii) benefits from practitioner feedback, and (iii) was able to successfully identify COVID-19 clusters from ED notes at early stages.

In [ 43 ], the authors applied a hierarchical model to classify patient records over two binary decisions: infection/no infection and known/unknown infection. They initially performed information extraction on the unstructured text, grounding the detected entities into the British Medical Journal's Best Practices knowledge base. The obtained features were combined with structured patient data to create a case vector. The TextCNN-Attention and LightGBM algorithms were then applied in turn to classify cases into the predetermined classes. Finally, the Knox method was applied to the infectious cases to analyse the grouping according to time and spatial characteristics. The resulting system was evaluated over a large dataset of 93,555 EHRs, containing 37,422 cases of infectious diseases. The evaluation showed that their method compares positively against other algorithms, with high average negative predictive value (82.6%), and reaching an average prediction accuracy of 90.4% for emerging infectious diseases.

In [ 41 ], the authors extend a tracker for ILIs for the detection of outbreaks of unknown illnesses by relying on time series analysis. The unknown diseases are flagged when symptoms deviate from the expected data, and they do not fit any known disorder. Empirical P-values are computed to represent deviations from the prevalence of known diseases in the previous 60 days using the findings from EHRs (applying the Topaz parser). When the P-value is less than 0.01, an “unusual day” is flagged; a sequence of “unusual days” indicates the possible presence of an emerging disease. Using this approach, they show empirically that they were able to retrospectively identify a new Enterovirus D68 outbreak that was also detected by the Centers for Disease Control and Prevention (CDC).

The number of studies on emerging diseases is also small, but the results show the feasibility of different NLP approaches for this kind of task.

### 3.4. Veterinary Medicine

NLP for animal diseases is gaining growing attention, due in part to the accessibility of animal health records. In veterinary epidemiology, recent reviews mention the importance of NLP for outbreak detection [ 18 20 ], although the applied NLP methods are typically not described in detail. Standalone research papers in this area have focused on diverse tasks: automatic labelling of disease codes in veterinary health records, analysing veterinary records to extract drug prescriptions, and identifying exposure pathways for zoonotic diseases.

In [ 62 ], the authors built PetBERT, a LLM trained on over 500 million words from 5.1 million veterinary EHRs across the UK. They fine-tune PetBERT-ICD for the automatic labelling of disease codes in veterinary health records with good performance. These resources have the potential to be applied to infectious disease surveillance.

[ 45 ] also uses LLMs over veterinary records for extracting essential prescription attributes such as dose, duration, active ingredient, and indication. They focus on antimicrobial resistance, and compare different prompting approaches to a baseline BERT-based fine-tuned model (VetBERT). They apply functional prompting, which combines probabilistic outputs with rule-based calculations, enforcing data types for extracted prescription attributes, and executing formulaic calculations for dose and duration. They report that functional prompting achieves high performance, well above the baseline model.

Related research is also carried out on zoonotic diseases, which are infectious diseases that can be transmitted between animals (vertebrates) and humans. [ 46 ] implemented an NLP pipeline based on topic modelling over open text questionnaires of Q fever notifications. In this work, the authors rely on the extracted topics to identify demographic-specific transmission pathways, study spatial patterns in their distribution and explore variations in animal exposures across topic groups. They apply Markov Random Fields to identify potential animal exposures, and regression models to associate exposures and hospitalisation probability. They conclude that surveillance combined with text modelling is useful for identifying exposure pathways.

### 3.5. Clinical NLP cf. NLP for Infectious Disease surveillance in Clinical data 

Our search identified numerous works that are related to the defined scope of survey (NLP over EHRs for the processing of infectious diseases), but are not strictly within. We introduce them here briefly, in order to highlight the broader application context that the relevant work sits within, and recent related directions that are emerging.

Clinical NLP researchers often aim to develop general solutions to problems. Hence, we found work that is not specific to infectious diseases, but potentially applicable for infectious disease surveillance. In [ 53 ], a hybrid system was developed that leverages LLMs and aims to identify diseases mentioned in radiology reports, framed as either multiple-choice disease classification or open-ended disease mention detection. This work relies on a rule-based system and GPT-4 for generating weak (synthetic) labels, which are used to fine-tune LLaMA 3.1-8B. The evaluation shows that the fine-tuned models can improve the baseline performance. This approach offers a scalable solution to deploying NLP in surveillance systems.

Exploring a more general solution to processing clinical text, in [ 63 ] diverse biomedical semantic textual similarity and concept representation tasks are explored using LLMs. The authors achieve good performance over clinical text on entity linking, i.e. mapping of disorder terms or other clinical concepts to abstract ontology identifiers. This would be valuable for surveillance workflows to support data normalisation. The authors leverage GPT-3.5's latent biomedical knowledge to generate definitions of UMLS and SNOMED-CT concepts. Subsequently, they build on their previous work [ 64 ], using contrastive learning to learn representations of pairs of these ontological entities and their LLM-produced definitions. They reduce the dimensionality of the embedding space through distillation, using the same (non-fine-tuned) base model to perform regression on projections of the average of entity-definition pair embeddings, to prevent catastrophic forgetting from repeated exposure to contrastive training. Finally, they perform parameter averaging from the ensemble of distilled models, in the model soup paradigm [ 65 ].

We also identified work that describes syndromic surveillance, i.e. surveillance of symptoms and syndromes, rather than infectious disease surveillance. In [ 50 ], the authors compare LLMs and other ML approaches for the identification of febrile convulsion cases in ED triage texts. The applied techniques include standard Neural Networks and other classifiers, as well as the fine-tuned RoBERTa-large-PM-M3-Voc model [ 66 ]. They evaluate the performance, concluding that LLMs can perform effectively in this setting after data augmentation and the addition of negative examples.

A final group of papers relates to the use of LLMs for tasks such as infectious disease diagnosis or clinical management, rather than surveillance. In this use, LLMs are applied in a predictive or generative capacity, rather than to support text analysis or extraction. [ 47 ] analyses the quality and safety of the suggestions made by ChatGPT-4 (Chat Generative Pre-trained Transformer 4) in real-life clinical practice for the management of patients with positive blood cultures. The authors use prompt engineering and request treatment plans for patients, providing demographics, medical history, clinical features, and results from relevant tests, including blood culture results. Overall, management plans were considered optimal for only one patient, satisfactory in 17 (39%), and as harmful in seven (16%). The authors conclude that this approach has severe limitations for current practice.

ChatGPT-4 was also applied to the detection of catheter-associated urinary tract infections (CAUTIs) in [ 49 ]; in this case, the results were more promising. Synthetic datasets were manually created, representing structured EHRs with demographics, body temperature, urine culture results, and calendar dates of admission and catheterisation (i.e. insertion and removal). The authors used prompt engineering to query ChatGPT-4 for different tasks: detection of patients with CAUTIs, retrieval of patient and catheter days, and calculation of the catheter utilisation ratio. The evaluation on test data achieved precision of 92% and recall of 91% on positive patient identification. The authors also claim success on extracting other relevant fields, although these were not part of the evaluation.

#### Datasets and Resources 

In this section, we provide an overview of the key datasets and resources used in NLP applications for infectious disease research. Table 2 presents a comprehensive list of these datasets, detailing their names, targeted infectious diseases, sourced clinical documents, number of records, and data modalities. These datasets cover a wide range of infectious diseases, encompassing both common and rare diseases. The sources of these data are diverse, including real-world hospital data, but also synthetic data generated by LLMs. The data modalities include text, images, and numerical data, enabling their application across a wide range of NLP tasks.

Named Entity Recognition (NER) and Relation Extraction (RE) are fundamental NLP tasks that involve identifying and classifying entities and their relationships within clinical texts. Datasets such as MedNLI, the PharmaCoNER corpus, and the CHIFIR dataset are instrumental in training models to extract meaningful medical information from unstructured data.

Text classification tasks aid in disease diagnosis by utilising datasets such as WCM, VA CDW, and Symptom2Disease, which provide extensive textual data for training classification models to predict patient outcomes and diagnose diseases. Besides, chest-related datasets including PadChest, Chest ImaGenome, CheXpert, and MIMIC-CXR, integrate radiology images with corresponding textual reports, facilitating the development of multimodal classification models for respiratory infections such as pneumonia [ 82 ].

Report generation is another critical application, aiming to automate the creation of clinical reports from radiology images to reduce the burden on physicians [ 83 ]. Datasets like MIMIC-CXR and PadChest, which contain both text and image data, are essential for training deep learning models to generate accurate radiology reports based on chest images [ 84 ].

Disease surveillance and event extraction are crucial for timely detection and monitoring of infectious diseases. The NNDSS public datasets provide extensive EHRs and numerical records that support large-scale epidemiological studies and trend analysis for diseases such as influenza and salmonella. The CHIFIR dataset facilitates the automated surveillance of invasive fungal infections by analysing cytology and histopathology reports in clinical data [ 40 ].

## 4. Conclusions

NLP techniques over EHRs have been widely studied in recent years, and one of the most relevant target domains is infectious diseases. The COVID-19 pandemic created a surge of interest in infection surveillance and modelling, and this has continued to grow, with research also now addressing other diseases such as invasive fungal infections or pneumonia. The applied techniques vary significantly in complexity, from rule-based techniques to fine-tuned LLMs, but in most cases the research shows that NLP methods can provide results useful for integration in medical surveillance workflows. Recent experiments with LLMs have already shown potential for multiple applications, although there are also negative results when the model is not adapted to the target task [ 47 ].

The need to target emerging and unknown diseases has also been studied in recent work. As the COVID-19 pandemic showed, surveillance systems need to adapt to conditions that have new and even evolving symptomology. Specific techniques are required to dynamically adapt to this shifting terminology. Recent work has explored hierarchical models, topic models, and time series analysis, showing strong performance. Further efforts to detect emerging phenomena through shifting descriptors evident in EHRs may prove useful, providing a hallmark of an emergent event [ 10 ].

Another relevant subdomain is that of animal diseases. Access to veterinary records for research can be more easily obtained, and this is one of the incentives to study this area. We identified articles relying on LLMs and topic models to effectively analyse these records. There was also one recent study on zoonotic diseases over Q fever notifications that showed the potential of NLP methods to monitor these disorders in large populations.

Clinical NLP that is not specifically targeted to infectious diseases has also seen a surge of research. Advances in this field could pave the way to more general solutions, and we have included articles covering a large variety of tasks: identification of disease mentions, semantic textual similarity, concept extraction, syndromic surveillance, and treatment planning. Some of these works are evaluated on different benchmark datasets that are not exclusively EHRs, to illustrate the portability of the different methodologies.

Highlighting the interest in this area, multiple annotated datasets are now available for research, with traditional NLP tasks, NER, RE and Text Classification being the most common. One of the main types of EHRs in this domain are radiology reports and they include multimodal data, where the image of the scan is given alongside the report. These collections are used to develop and evaluate automatic generation of reports, which is a critical task in medical workflows for infectious diseases. Another recent development with regards to resources is the use of LLMs for generating datasets. We include some recent corpora developed using these methods.

All in all, NLP for infectious diseases is a very active area in health informatics, and multiple research initiatives illustrate the need for incorporating robust NLP techniques when dealing with the prevalent unstructured data in this domain. General advances in the sophistication of NLP techniques arising from language models, including recent clinically-tailored ones learned from healthcare data such as CLMBR [ 85 ], may offer new opportunities to enable improved concept and relation extraction from clinical texts, in turn laying the foundation for improved strategies for transforming unstructured data into actionable information for infection detection, surveillance, and prediction. It is worth noting, however, that generative LLMs are not a panacea – they still have a long way to go before they can be safely and effectively leveraged in the clinical domain [ 86 ].

## Footnotes

1
https://www.who.int/our-work/communicable-and-noncommunicable-diseases-and-mental-health

## Footnotes

2
https://anthology.acl.org

## Footnotes

3
https://notebooklm.google/

## Footnotes

4
https://exa.sh/search

## Footnotes

5
https://www.emro.who.int/health-topics/emerging-diseases/index.html
